# A Systematic Review of Sexual and Reproductive Health Knowledge, Experiences and Access to Services among Refugee, Migrant and Displaced Girls and Young Women in Africa

**DOI:** 10.3390/ijerph15081583

**Published:** 2018-07-26

**Authors:** Olena Ivanova, Masna Rai, Elizabeth Kemigisha

**Affiliations:** 1 Division of Infectious Diseases and Tropical Medicine, Medical Centre of the University of Munich (LMU), 80802 Munich, Germany; masnarai11@gmail.com; 2 Faculty of Interdisciplinary Studies, Mbarara University of Science and Technology, P.O. Box 1410, Mbarara, Uganda; ekemigisha@must.ac.ug

**Keywords:** sexual and reproductive health, adolescent, refugee, migrant, young women, knowledge, access, experiences, systematic review, Africa

## Abstract

Adolescent girls and young women are an overlooked group within conflict- or disaster-affected populations, and their sexual and reproductive health (SRH) needs are often neglected. Existing evidence shows that forced migration and human mobility make girls and women more vulnerable to poor SRH outcomes such as high risk sexual behaviors, lack of contraception use, STIs and HIV/AIDS. We performed a systematic literature review to explore knowledge, experiences and access to SRH services in this population group across the African continent. Two databases (PubMed and Web of Science) were searched and from 896 identified publications, 15 peer-reviewed articles published in English met the inclusion criteria for this review. These consisted of eight applied qualitative, five quantitative and two mixed-method study designs. The quality of the studies was evaluated by the mixed-methods appraisal tool (MMAT) using scores in percentages (0–100%). Available evidence indicates that knowledge of young women and girls regarding contraceptive methods, STIs and HIV/AIDS are limited. This population group often experiences gender-based and sexual violence and abuse. The access and availability of SRH services are often limited due to distances, costs and stigma. This review demonstrates that there is still a dearth of peer-reviewed literature on SRH related aspects among refugee, migrant and displaced girls and young women in Africa. The data disaggregation by sex and age should be emphasized for future research in this field.

## 1. Introduction

Every year, thousands of people around the world flee their homes to escape conflict, disasters and violence within their own country or by crossing international borders. During these humanitarian emergencies, refugees and internally displaced persons (IDPs) suffer a great pressure which affects their health. Adolescent girls and young women (10–24 years old) are an overlooked group within conflict- or disaster-affected populations, and their sexual and reproductive health (SRH) needs remain largely unmet [1]. As such, the synergy of crisis as well as the neglect of SRH needs, increases vulnerability of adolescent girls to unwanted pregnancies, HIV and sexually transmitted infections (STIs), maternal death and sexual violence [2,3,4]. Adolescent girls in humanitarian settings experience increased exposure to early and forced marriage, coerced sex and early childbearing, as well as increased risk-taking associated with gender roles in family circles [5]. The girls in refugee camps are also vulnerable to sexual exploitation and trafficking [6].

Use and knowledge of SRH services and commodities, including family planning, are also often low among women and girls in humanitarian settings. One of the very few studies on this subject by McGinn et al. demonstrated that knowledge and use of modern contraceptive methods was low among married or in-union women of reproductive age in six reproductive health program locations (North Darfur, West Darfur, South Darfur, Southern Sudan, Northern Uganda and Eastern Congo) in three conflict-affected countries—Sudan, Uganda and the Democratic Republic of Congo [7].

The access to SRH services is challenging for young women and girls living in refugee settlements or dispersed across host countries. The minimum health care package in humanitarian setting requires inclusion of sexual and reproductive health services [8]. A systematic review by Casey et al. established that implementation of SRH programs is possible in a humanitarian setting, however their utilization depends on their quality [9]. Furthermore, the review highlighted minimal provision of adolescent SRH services [9]. In 2004 a global evaluation of reproductive health services for refugees and IDPs concluded that most people affected by conflict lack adequate SRH care [10]. The evaluation found that contraceptive methods offered were frequently limited to pills and condoms. Long-acting contraceptives or permanent methods were rarely offered, and for all methods, supplies were often not reliable. The evaluation also pointed that adolescents were often underserved [10]. Since then, much progress has been made in the field of SRH in crisis settings in terms of policy, guidance and practice and steps have been taken toward integrating SRH into humanitarian response including increased program funding to implement SRH services [11]. Nevertheless, most conflict-affected girls and women still do not have adequate access to SRH services with family planning services often being particularly neglected. From 2012 to 2014, the Inter-agency Working Group (IAWG) undertook a second global evaluation of reproductive health in humanitarian settings. One of the series of reviews and articles concluded that although services are being provided, the availability of good quality SRH services was inconsistent across different settings. There was still a limited knowledge of available services and socio-cultural barriers to accessing them among communities [12]. 

Although, the research on the SRH of conflict- and disaster-affected populations is gaining attention globally, there is very scarce information on the needs and experiences of young refugees and IDPs in humanitarian settings, who are likely to be at particularly high risk of adverse SRH outcomes. Enhancing the weak evidence base on needs and experiences of crisis-affected girls and women will provide the tools for the research community and humanitarian actors to identify better ways to serve the SRH needs of these population groups. This systematic review aims at exploring and synthetizing the available evidence on sexual and reproductive health knowledge, experiences and access to services among refugee, migrant and displaced girls and young women in Africa.

## 2. Materials and Methods

A mixed methods systematic review, exploring qualitative and quantitative data, was conducted to assess our aim. Sandelowski et al. identified three general frameworks through which to conduct mixed methods systematic reviews—segregated, integrated, and contingent [13]. We have applied the integrated methodology for our review. In integrated designs, the methodological differences between qualitative and quantitative studies are minimized and the studies are grouped for synthesis by results addressing the same research questions, or describing the same aspects of a target phenomenon [13].

### 2.1. Search Strategy

We adhered to the Preferred Reporting Items for Systematic Reviews and Meta-Analyses (PRISMA) guidelines for systematic reviews [14]. Two electronic databases—PubMed and Web of Science were searched. Search terms for sexual and reproductive health covered the topics relevant to conflict-affected situations, e.g., sexual violence, maternal health, as well as the topics of a particular relevance to young girls and women, e.g., sexuality education, teenage pregnancies, etc. Our choice was also guided by the SRH definitions from the International Conference on Population and Development (ICPD) in 1994. Search terms are described in Table 1. Data search was performed between the end of March and the beginning of April 2018. In addition, we completed a manual search of the reference lists of relevant articles. All records were exported into Mendeley—an online bibliographic management program produced by Elsevier. After we removed the duplicates, titles and abstracts were screened for inclusion.

### 2.2. Study Selection

**Figure 1:**
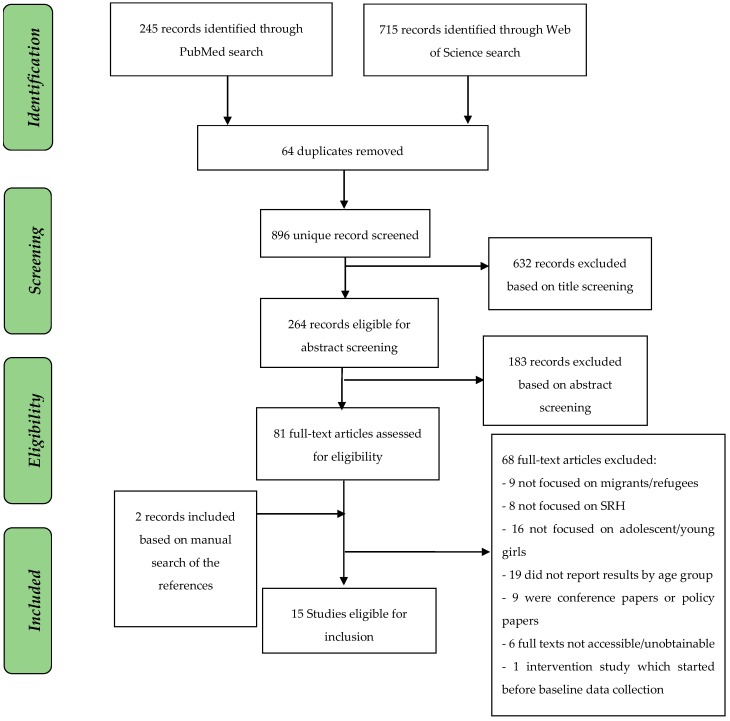
PRISMA Flow Diagram.

This review was limited to peer-reviewed, full-text articles published in English before March 2018. While we initially planned to search the grey literature, our time and financial resources did not permit us to explore this literature. Studies providing insufficient information, for example letters, abstracts or conference papers, were excluded. Study population of interest included refugee, migrant or displaced women and girls. For refugee, migrants and internally displaced populations (IDPs) we applied the official definitions of The United Nations (UN) and International Organization for Migration (IOM). Only studies which included results on adolescent and young women (10–24 years old) were considered. The UN define adolescents as individuals being 10–19 years old and youth as those persons between the ages of 15 and 24 years. We excluded studies which reported results on SRH of migrants and refugees without sex and age disaggregation. Only studies, which were conducted in African countries were included. Studies related to refugees of African origin settled in other region other than Africa were excluded. Details of the study selection are summarized in Figure 1.

### 2.3. Critical Appraisal

Two researchers independently reviewed the full texts for quality and suitability. Eligible papers then underwent a quality appraisal using the MMAT tool [15]. This tool was designed for systematic mixed studies reviews. The tool helps to examine the appropriateness of the study aim, adequacy and methodology, study design, data collection, study selection, data analysis, presentation of findings, author’s discussions and conclusions. For each of the included studies, the relevant four quality questions were asked corresponding to the study type, e.g., qualitative, quantitative descriptive or mixed methods. The studies were scored using percentages (0–100%). Any discrepancies were discussed until a consensus was reached.

### 2.4. Data Extraction and Analysis

Two authors independently read all included articles and completed a data extraction form created to gather the following: authors, study setting, main study objectives, study population, study design and study findings. Due to the inclusion of studies using qualitative and quantitative design, conducting a meta-analysis of the data was not appropriate. A descriptive narrative synthesis was chosen as the most relevant and suitable method of data synthesis for this review. The results are supported with original quotations and examples.

## 3. Results

### 3.1. Study Characteristics

From 960 records, 15 studies met the inclusion criteria. They were conducted in nine African countries which included Uganda, Ethiopia, DR Congo, Somalia, Kenya, Nigeria, Djibouti, Rwanda and Sierra Leone [6,16,17,18,19,20,21,22,23,24,25,26,27,28,29]. These consisted of eight applied qualitative, five quantitative and two mixed-method study designs. Three studies were performed exclusively with young girls and women of age groups 10–24, 12 other studies included them as one of the groups in total population, e.g., among women of reproductive age or adolescents including boys and girls. One study [16] reported the results before and after the intervention. For the purpose of this review we have used the baseline data from the above mentioned study on HIV/STDs knowledge and attitudes among young displaced girls. In the case of the study performed by Stark et al. [26] we only used the qualitative data for this review because the quantitative data were previously reported within a bigger sample in Stark et al. [25]. Tanabe et al. [28] focused on refugee adolescent girls living with disabilities. Four studies also included data collected in Asian or Middle East countries apart of African countries [27,28,29]. Further details on the methodology and the main objectives of the included studies are presented in Table 2.

### 3.2. Sexual and Reproductive Health Knowledge

Six articles reported on knowledge and misconceptions regarding contraception, HIV/AIDS and STIs [16,18,19,21,27,28]. Overall, the knowledge of refugee and displaced young women and girls on the full range of contraceptive methods were limited. Also, the adolescents were less aware about modern methods of contraception than the adults in the same setting [27]. Nevertheless, the young participants could name at least one method of contraception [21,28]. Two studies demonstrated that the misinformation about contraceptive methods is widespread preventing young women and girls from using them [18,21]. Knowledge of routes of HIV transmission and HIV prevention were also limited [16,19]. Casey et al. [16] demonstrated that 25% of female youth did not know any correct route of HIV transmission, while 28.7% did not know any effective means of avoiding HIV. Harrison et al. [19] showed that comprehensive correct knowledge of HIV/AIDS among refugee females in Uganda aged 15–24 was 33.5%. Casey et al. [16] also found low knowledge among female youth in Sierra Leone regarding STIs —33.2% of participants did not know any signs of STIs.

### 3.3. Sexual and Reproductive Health Experiences and Practices

All studies shared findings on the experiences and practices of female youth including contraceptive use, gender-based and sexual violence, child marriages, transactional sex, puberty changes and decision-making. Half of the studies reported on the prevalence of and fears related to gender-based or sexual violence and abuse in humanitarian settings [6,17,22,23,25,26,29]. In the study of Feseha et al. [17] the magnitude of partner’s physical violence in the last 12 months in the Schimelba refugee camp in northern Ethiopia among girls aged 15–19 was 10% and 41% among girls 20–24 years old. High prevalence of violence (51.6%) among conflict-affected girls 13–19 years old was also reported in DRC and Ethiopia by Stark et al. [25]. The qualitative study from Iyakaremye and Mukagatare [6] performed with adolescents in Kigeme refugee camp in Rwanda found that rape, unwanted physical touching, sexual exploitation, commercial sex, early marriage and trafficking were the main forms of sexual abuse. Forced sex and rape among young women were also reported in the qualitative study of Whelan et al. [29]. Sexual violence was also mentioned by the majority of girls in Ethiopia and DRC and was considered to be the most serious type of violence affecting the community: *“sometimes there are drunk men who rape girls along the road”* (age 15, Ethiopia) [26]. This sexual abuse leaves girls with consequences such as unwanted pregnancies leading to unsafe abortions, rejection and harassment in their families [6]. The topic of child/early marriages was raised in qualitative studies and it was seen as an important concern among young adolescents [6,22]. It was perceived as a barrier to pursue education and often resulted in early pregnancies: “*(for) the ladies, it’s possible that they may not continue with the education and they may end up in early marriage*” (girls, 13–14 years old). [22].

Transactional and commercial sex, so called “survival sex” in exchange for goods, food, menstrual hygiene products and money was highlighted in some studies [6,19,21,23,29]. “*Many girls in this camp sleep with men in order to survive. We are here in Nigeria with nothing and nobody to help us and we have to survive*” (18 year old young women) [21]. Harrison et al. [19] found that 66.7% of sexually active females aged 15-24 had a transactional partner in the past 12 months. At the same time only 16.7% of them used a condom during the last sexual encounter. The low condom use was also documented in three other studies [16,21,27]. This also applies to other contraceptive methods. The main reasons identified for non-use were partner refusal, misinformation, religion, fear of side effects and stigma. One girl from the Oru refugee camp in Nigeria commented: “*I like using condom during sex but I didn’t use it the last time I had sex because I didn’t want to buy it in the camp. I don’t want anyone to think I’m a prostitute. I remember the last time I went to buy condom and pills in a chemist in the camp; I was embarrassed by some women. They asked me what a young girl like me wanted to do with condom and pills. I was so ashamed that I had to lie that someone sent me. So, I prefer buying it outside the camp, a little bit far away, where no one knows me.*” [21]. Only one study explored body/puberty changes in refugee adolescent girls which is an important aspect of adolescents’ sexual and reproductive health [20].

### 3.4. Access to Sexual and Reproductive Health Services, Commodities and Information

Several studies addressed the access to SRH services and information [18,20,21,22,23,27,28,29]. Girls and young women stated parents (mostly mothers), siblings and peers as main sources of SRH information on pregnancy, puberty, menstruation, etc. [20,22,23]. Lack of information regarding different areas of SRH was documented in the above studies. For example, the majority of Somali girls (95.2%) indicated having learned about body changes before they occurred, however 66.8% would like to have had more information and very few girls of age 10-14 reported learning about pregnancy (18.2%) [20]. Patel et al. [23] in a qualitative inquiry found that many participants reported access to information on HIV/AIDS and very few (6%) had access to information on contraception, sexuality, abortion and pregnancy. The same study showed that girls younger 18 years old face problems to access information about family planning: “*When they (family planning services) are teaching about condoms, they usually restrict it to people of 18 years and above. They are the ones who are advised to use it. The use of family planning is for married women (those with husbands) not for girls. . . young girls in the ages of 12–14 years don’t have any knowledge about condoms.*” (adolescent girl) [23].

Adolescent girls also lacked access to SRH commodities including menstrual hygiene products and contraception. Only 61.5% of Somali girls in Kobe refugee camp stated having sufficient access to soap and water, but less than one girl in five (19.2%) had access to cloth/pads to use during menstruation [20]. Three studies documented a limited access to contraceptive methods including condoms [21,23,27]. A high proportion (60 to 91%) of adolescent girls found it difficult to obtain contraceptives in the camps [21,23]. The main barriers were distance to the health care facilities, costs, lack of knowledge about sources of contraception, stigma and judgmental attitudes. For example, adolescent girls in Nakivale camp, Uganda reported that they often find the condom dispensers in the settlement empty [27]. Costs and distance to the health care services were highlighted in the study of Okanlawon et al. [21]: “*Before, contraceptives like pills, IUD, injectables,* etc.*, were free in this camp, but since the camp clinic was closed in 2007, we now go to hospitals in the surrounding community to receive the service. It’s no longer free. Some of us like to use it, but we can’t get it in the camp. That’s the problem.*” (24 years old young women).

The last point we would like to highlight is the quality of provided SRH services. Refugee women and girls complained about lack of facilities for STI testing, lack of acceptable and affordable contraceptives, stock outs, long waiting times, language barriers and discrimination [21,27,28,29].

### 3.5. Criticial Appraisal of Included Studies

The majority of studies (10 of 15) scored 75% and more, five other studies scored 50% and no study was assigned less than 50% (Table 2). Overall, we could conclude that the quality of included articles was from moderate to high.

## 4. Discussion

The aim of this review was to combine the existing evidence on SRH knowledge, experiences and access to services of young women and girls aged 10–24 in crisis and humanitarian situations. Such studies were largely absent and thus this review pointed out the lack of available peer-reviewed literature on SRH of girls and young women in the African region, where multiple conflicts are ongoing for decades, putting their health, including SRH at risk. To summarize, the young women and girls are lacking knowledge on SRH issues; access to this information is often hindered because of many different factors including stigma related to young age; access to SRH services and commodities is challenging because of distance, costs and quality.

The studies in this review show us the limited SRH knowledge and awareness among adolescent girls and the misconceptions about the contraceptives which cause the adolescents to refrain from using them. As a result, insufficient uptake of family planning contributes to morbidity and mortality in women and girls of reproductive age via pregnancy-related complications and deaths [30]. The studies have also explored the access to and sources of SRH knowledge and education in humanitarian settings but the sources preferred by the adolescent for obtaining SRH information has not been very well documented. An effective way of educating the adolescents about SRH would be through the sources they prefer.

The studies show high prevalence of sexual violence, coerced sex, transactional sex and other forms of sexual exploitations. In a few studies, this sexual harassment or physical violence may be instigated by own family members or partner [17,25]. Sexual exploitation by family members or close relatives may be quite difficult to assess as the adolescents may not be willing to open up to report such incidences. However, we believe that quite a large number of girls are suffering from sexual exploitations and harassments by their family members and close relatives apart from their partners. This finding has been reported previously in other studies in Africa in non-humanitarian settings [31]. These cases of sexual exploitations also need to be extensively studied.

In addition, we would like to discuss the main points which could help to strengthen future data collection in this field. Firstly, although many identified studies report on SRH of refuges and migrants, few of them disaggregate findings by age and sex of respondents. During the full text review and data extraction process we faced challenges to separate results for youth and for female participants, excluding several studies from this review. Some of the included studies also stressed the necessity to perform target research in this age group [20]. A number of studies have been performed on SRH of women of reproductive age. The results which are not segregated by age, may confuse the current situation and needs of the adolescents with those of the women of higher reproductive age.

Secondly, multi-setting studies provide reach and comparable information across different humanitarian settings. However, here the disaggregation of results by setting plays an important role as the findings of one humanitarian settings cannot be generalized onto the other due to the differences between the settings. The differences may exist due to cultural values and background of the refugees and the infrastructural and economic differences of the hosts.

### Limitations

This systematic review has a number of limitations. Only studies written in English were included. The MMAT appraisal tool for mixed-method research was used to assess the quality of reporting in the studies, but more specialized quality assessment tools such as the Cochrane Collaboration’s tool for assessing risk of bias would have provided more in-depth reviews of quality. Unfortunately due to time constraints and funding, this review did not include grey literature, such as UNFPA and UNHCR reports and studies conducted by non-governmental organizations working in humanitarian settings. These studies could have provided valuable data on SRH indicators and situation of girls and women affected by conflicts and disasters. Although PubMed and Web of Science are the most often used search databases, we might have missed some relevant studies included to other databases, e.g., Global Health or EMBASE. Some of the peer-reviewed studies which could not be accessed, were also not included.

## 5. Conclusions

Results of this review demonstrate the gaps in the existed evidence on SRH of migrant, refugee and displaced girls and women living in Africa. The necessity of disaggregation by sex and age should be addressed in future research. Targeting young refugee, displaced and migrant adolescents of 10–14 years old is very important enabling the complexity of body and physiological changes and the paucity of information from this age and population group to be taken into account.

## Figures and Tables

**Table 1:** Search terms used in PubMed and Web of Science.

Category	Search Terms Combined with AND
Age Group	youth OR teenager OR teen OR girl OR young female OR adolescent OR woman OR young woman OR women OR young person OR adolescence OR female OR reproductive age
Age	10 to 24
Status	refugee OR migrant OR displaced OR displaced person OR foreigner OR immigrant OR ethnic minority OR indigenous OR internally displaced OR asylum
SRH topics	sexual OR sexual health OR reproductive health OR early marriage OR child marriage OR female genital mutilation OR cutting OR female circumcision OR circumcised OR sexual behavior OR sexual experience OR sexual activity OR early sexual debut OR sexual initiation OR menstruation OR menstrual hygiene OR contraception OR family planning OR pregnancy OR antenatal OR birth OR post-natal OR sexually transmitted infection OR STI OR sexual intercourse OR HIV OR violence OR sexuality education OR reproduction OR sexual well-being OR condom OR human immunodeficiency virus OR AIDS OR sex education OR sex OR relationship OR physical relationship OR sexual coercion OR rape OR sexual violence OR sexual abuse OR abortion OR maternal health OR fistula OR motherhood OR gender OR forced sex OR intimate partner violence OR gender based violence OR transactional sex OR sex work OR HPV OR cervical cancer
Outcome	need OR unmet need OR access OR knowledge OR availability OR experience OR awareness OR perception
Countries/regions	Africa OR Algeria OR Angola OR Benin OR Botswana OR Burkina Faso OR Burundi OR Cameroon OR Cape Verde OR Central African Republic OR Chad OR Comoros OR Congo OR Democratic Republic of the Congo OR Cote d'Ivoire OR Djibouti OR Egypt OR Equatorial Guinea OR Eritrea OR Ethiopia OR Gabon OR Gambia OR Ghana OR Guinea OR Guinea-Bissau OR Kenya OR Lesotho OR Liberia OR Libya OR Madagascar OR Malawi OR Mali OR Mauritania OR Mauritius OR Morocco OR Mozambique OR Namibia OR Niger OR Nigeria OR Rwanda OR Sao Tome OR Senegal OR Seychelles OR Sierra Leone OR Somalia OR South Africa OR Sudan OR South Sudan OR Swaziland OR Tanzania OR Togo OR Tunisia OR Uganda OR Zambia OR Zimbabwe, NOT (Europe OR EU OR European Union OR Australia OR US OR New Zeeland OR United States OR France OR Greece OR Italy OR Austria OR Belgium OR Latvia OR Bulgaria OR Lithuania OR Croatia OR Luxembourg OR Cyprus OR Malta OR Czech Republic OR Netherlands OR Denmark OR Poland OR Estonia OR Portugal OR Finland OR Romania OR France OR Slovakia OR Germany OR Slovenia OR Greece OR Spain OR Hungary OR Sweden OR Ireland OR United Kingdom OR UK OR America OR Asia OR Brazil OR South America OR Latin America)

**Table 2:** Characteristics of studies included in the review.

N	Author	Year	Country	Population	Age Group Included in the Review	Design	Main ResearchObjective/Aim	Quality of Studies (MMAT)
1	Casey et al. [16]	2006	Sierra Leone	Youth displaced by conflict (244 female and 293 male participants)	15–24	Quantitative Cross-sectional Survey (baseline data)	Explore the HIV/AIDS/STD knowledge, attitudes and behaviors of youth	50%
2	Feseha et al. [17]	2012	Ethiopia	Refugee women (422) including 40 girls of 15–19 years old and 156 of 20–24 years old	15–24	Quantitative Cross-sectional Questionnaire	Assess the magnitude of intimate partner physical violence and associated factors among women	100%
3	Gure et al. [18]	2015	Somalia	Unmarried girls in displacement camps (5) among total sample of 21 married and unmarried women	18–20	Qualitative Cross-sectional FGDs	Explore women’s knowledge of, experiences with, and need for reproductive health services	50%
4	Harrison et al. [19]	2009	Uganda	Refugees and host communities (1600) with 120 (19.8%) of total female refugees (607) being girls of age 15–19 and 100 (16.6%) of 20–24 years old	15–24	Quantitative Cross-sectional Standardized behavioral surveillance survey (BSS)	Provide data on HIIV related knowledge, attitudes and behavior among refugees and surrounding hosts populations to allow for targeted HIV interventions	100%
5	Iyakaremye and Mukagatare [6]	2016	Rwanda	Adolescent girls from DRC(10) in total sample (17) of boys, mothers, fathers and staff	Adolescent girls age not mentioned	Qualitative Cross-sectional Interviews and FGDs	Explore the experience of sexual abuse of adolescent girls in refugee camp	75%
6	Kågesten et al. [20]	2017	Ethiopia and Thailand	Young Somali adolescents (406) in Kobe refugee camp, from which 214 (52.7%) were girls; and young adolescents from Myanmar (399)	10–14	Quantitative Cross-sectional Household survey	Describe transition into puberty and access to SRH information among very young adolescents in humanitarian setting	75%
7	Okanlawon et al. [21]	2010	Nigeria	Youth in Oru refugee camp (116 female of total 208)	10–24	Qualitative and Quantitative Cross-sectional Self-administered questionnaire, in depth interviews and FGDs	Examine the perceptions, beliefs, knowledge and attitudes of refugee youths towards contraceptive use and also the access to and use of contraceptives in this refugee camp	Qualitative—75% Quantitative—50% Mixed—50%
8	Ortiz-Echevarria et al. [22]	2017	Ethiopia	Somali refugees and host community (126–32 adults and 94 adolescents including 46 refugee girls)	10–16	Qualitative Cross-sectional FGD with community mapping and photo	Understand lived realities of very young adolescents in Kobe refugee camp, their health and development needs, expectations and goals	75%
9	Patel et al. [23]	2012	Uganda	Acholi girls (67) and adult women (65) in three displacement camps in Gulu district	14–19	Qualitative Cross-sectional In-depth interviews and FGD	Provide a better understanding of adolescent girl’s enhanced risk for HIV infection in conflict settings and to inform the development of appropriate sexual education and HIV prevention initiatives in this population group	100%
10	Schlecht et al. [24]	2013	Uganda	Displaced and refugee men and women from Uganda and DRC (133)	10–24	Qualitative Cross sectional FGDKey informant interviews	Describe the factors which contribute to early relationships and informal marriages in conflict and post-conflict settings	75%
11	Stark et al. [25]	2017	DRC and Ethiopia	Displaced, conflict-affected adolescent girls (1296)	13–19	Quantitative Cross-sectional Survey questionnaire using computer-assisted personal interview and computer-assisted self-interview	Assess the prevalence and related risk factors of physical, emotional, and sexual violence	100%
12	Stark et al. [26]	2017	DRC and Ethiopia	87 internally displaced adolescent girls from DRC and 78 Sudanese girls in Ethiopian refugee camps	10–19	Qualitative Cross-sectional Qualitative participatory mapping activity	Provide insight into assessing gender based violence from two methodological approaches	75%
13	Tanabe et al. [28]	2015	Kenya, Nepal and Uganda	352 refugee female and male participants from them44 adolescent girls with any type of impairment in Kenya and Uganda	15–19	Qualitative Cross-sectional Individual interviews and FGDs	Explore the specific risks, needs and barriers for persons with disabilities to access SRH services, and the capacities and practical ways through which the challenges could be addressed	75%
14	Tanabe et al. [27]	2017	Djibouti, Kenya, Uganda, Bangladesh, Jordan and Malaysia	Adolescents, women and men in refugee settings	15–19	Qualitative and Quantitative Cross-sectional Household survey, in-depth interviews, FGDs and facilities assessment	Document the knowledge of family planning, belief and practices of refugees, and the state of service provision	Qualitative—50% Quantitative—50% Mixed—50%
15	Whelan [29]	2007	Uganda, Yemen and DRC	Refugees (816) including sample of 78 girls from Uganda and DRC participating in FGDs	Adolescent girls age not mentioned	Qualitative Cross-sectional FGDs and interviews	Identify factors that facilitate or hinder access to, use of, and satisfaction with RH services in refugee settings	50%

## Abbreviations

AIDS	Acquired immune deficiency syndrome
HIV	Human immunodeficiency virus
IAWG	Inter-agency Working Group (IAWG)
IDP	Internally displaced people
FGD	Focus group discussion
MMAT	Mixed Methods Appraisal Tool
PRISMA	Preferred Reporting Items for Systematic Reviews and Meta-Analyses
SRH	Sexual and reproductive health
STI	Sexually transmitted infection
UNFPA	United Nations Population Fund
UNHCR	The United Nations Refugee Agency
